# Apoptosome-dependent myotube formation involves activation of caspase-3 in differentiating myoblasts

**DOI:** 10.1038/s41419-020-2502-4

**Published:** 2020-05-04

**Authors:** Mahshid H. Dehkordi, Amin Tashakor, Enda O’Connell, Howard O. Fearnhead

**Affiliations:** 1 Pharmacology and Therapeutics, School of Medicine, NUI Galway, Ireland; 2 Genomics and Screening Core, College of Science, NUI Galway, Ireland; 3 Present Address: Royal College of Surgeons, Dublin, Ireland

**Keywords:** Proteases, Cell biology

## Abstract

Caspase-2, -9, and -3 are reported to control myoblast differentiation into myotubes. This had been previously explained by phosphatidylserine exposure on apoptotic myoblasts inducing differentiation in neighboring cells. Here we show for the first time that caspase-3 is activated in the myoblasts undergoing differentiation. Using RNAi, we also demonstrate that differentiation requires both cytochrome *c* and Apaf-1, and by using a new pharmacological approach, we show that apoptosome formation is required. We also show that Bid, whose cleavage links caspase-2 to the mitochondrial death pathway, was required for differentiation, and that the caspase cleavage product, tBid, was generated during differentiation. Taken together, these data suggest that myoblast differentiation requires caspase-2 activation of the mitochondrial death pathway, and that this occurs in the cells that differentiate. Our data also reveal a hierarchy of caspases in differentiation with caspase-2 upstream of apoptosome activation, and exerting a more profound control of differentiation, while caspases downstream of the apoptosome primarily control cell fusion.

## Introduction

Cell death and differentiation are fundamental biological processes that are intimately connected at a molecular and cellular level. Diverse signals can induce apoptotic cell death by activating initiator caspases such as caspase-2, -8 and -9 which in turn activate effector caspases (caspase-3, -6 and -7) to kill a cell. The molecular pathways regulating caspase activity during apoptosis are known in some detail^1^. However, activated apoptotic caspases do not always kill cells; instead these “killers” can also play vital and non-cell death inducing roles in developmental and regenerative processes^2^. These alternative roles have been best demonstrated in model organisms such as *Drosophila melanogaster*^3^, but there are also examples of similar caspase roles in mammalian systems^4–7^. In contrast to the caspase-dependent cell death processes, the molecular mechanisms by which killer caspases control non-cell death processes are not well understood. In some non-apoptotic processes it appears that caspases are activated by the same molecules that cause apoptosis^3^. In other non-apoptotic processes the mechanism of caspase activation is not clear.

The differentiation of myoblasts into mature muscle is one example of caspase-dependent differentiation. In studies using both primary myoblasts and the C2C12 myoblast cell line, myoblast differentiation involves the activation of caspase-9^8^, -2^9^ and -3^10^. Myoblast differentiation is also blocked by anti-apoptotic members of the Bcl-2 family^8^ which prevent mitochondrial outer membrane permeabilization (MOMP) and release of cytochrome *c* from mitochondria during apoptosis. Spermatogenesis in *Drosophila* involves the mitochondrial pathway^3^, and although the data implicate the apoptotic mitochondrial (intrinsic) cell death pathway in muscle differentiation, other pathways can activate caspase-9^11,12^ and these could be important instead. Moreover, Bloemberg et al.^13^ dispute whether caspase-9 is involved in myoblast differentiation and have thrown doubt on whether the mitochondrial pathway has a role in muscle differentiation at all.

More uncertainty arises because nobody has yet demonstrated that increased caspase activity in a myoblast is required for that cell to go on to differentiate. Dying cells can provide caspase-dependent signals for tissue regeneration^14–17^. At the same time it has been reported that apoptotic myoblasts trigger differentiation in neighbouring healthy myoblasts^18^, so there is a possible explanation for the role of caspases in differentiation; one in which caspase-dependent apoptosis is a signal for tissue regeneration and there is no caspase activity in the cells that differentiate.

Here we have used a range of different approaches and addressed two key questions: Is caspase-3 activated in cells that go on to differentiate, and is the differentiation dependent on the mitochondrial death pathway? Our results show that caspase-3 is indeed activated in differentiating cells and by using a set of complementary approaches, we show that the differentiation is dependent on apoptosome formation. This led us to investigate events upstream of mitochondria and to test the idea that caspase-2-dependent cleavage of Bid was the trigger for differentiation.

## Materials and methods

### Inhibitors, plasmids and siRNA

Green-to-red FPX Caspase-3 reporter plasmids, GANES-DEVD-BNLS (50842), RANLS (50843) and single polypeptide FPX biosensor for caspase-3 (60883), and M50054 (ab145906) were purchased from Abcam (Cambridge, UK). MISSION® esiRNA’s targeting Apaf-1, Caspase-2 and Bid and control scrambled siRNAs and Q-VD-OPh (SML0063) were from Sigma-Aldrich (St. Louis, MO). Cycs Mouse siRNA Oligo Duplex (SR401266) was from OriGene (Rockville, MD). Plasmids, pcDNA3-Casp2-Flag (11811), pcDNA3-Casp2 C303A-Flag (11812), pCMV-BID (21131), pCMV-BID (D59E) (21133) and pCMV-tBID (21149) were purchased from Addgene. Details of primary and secondary antibodies, siRNAs and plasmids are provided in Supplementary Materials.

### Cell culture, myogenic differentiation and drug treatments

Mouse C2C12 myoblasts (Sigma-Aldrich) were maintained in Dulbecco’s modified Eagle medium (DMEM, Sigma-Aldrich) supplemented with 20% foetal bovine serum (FBS) and 1% penicillin/streptomycin (Sigma-Aldrich); referred to as growth medium or GM. To induce differentiation, the cells were seeded into µ-Plate 96 Well (Ibidi GmbH, Gräfelfing, Germany) at 2.8 × 10^4^ cells per well and incubated at 37 °C, 5% CO_2_ overnight. The next day, the cells were washed three times with Hank’s Balanced Salt Solution (HBSS, Sigma-Aldrich), and DMEM supplemented with 2% horse serum and 1% penicillin/streptomycin (differentiation medium; DM) was added. Differentiation was allowed to proceed for 3–5 days, at which point cell fusion was assessed. All drugs were prepared in working stocks of DMSO, and the final concentration of DMSO in all treatments was 0.1%.

### C2C12 transfection

C2C12 cells were transfected using Amaxa® Cell Line Nucleofector® Kit V (Lonza, Basel, Switzerland), program B-032. After 24 h transfected cells were seeded in µ-Plate 96-well plates (Ibidi GmbH) for further experiments.

### siRNA experiments

Reversed transfection of siRNAs (100 nM) was performed using Dharmafect-4 transfection reagent (Dharmacon Inc, Lafayette, CO), according to manufacturer’s instruction. The expression of proteins was assessed by immunoblotting after 72 h.

### Detection of caspase activity using a biosensor

The biosensor comprises a single polypeptide that contains the three domains. Interaction of a quenched green fluorescent protein domain (GA) with a second domain (B) generates green fluorescence. A third domain (RA) contains a quenched red fluorescent protein. Cleavage at a caspase-3 cleavage site between the GA and B domains releases the GA domain, favouring an interaction between RA and B. The result is that caspase-3 activity results in decreased green fluorescence and increased red fluorescence. U2OS cells were seeded into µ-Plate 96-well plates (Ibidi GmbH) and treated with etoposide (50 µM) for 24 h. Cells were imaged using an Operetta high-content imaging system (Perkin Elmer, Waltham, MA). Caspase-3 reporter transfected C2C12 cells were seeded into µ-Plate 96-well plates (Ibidi GmbH) and induced to differentiate. After 0, 24, 48, 72 or 96 h the cells were washed with HBSS and fixed with 4% paraformaldehyde at room temperature for 20 min. Cells were then examined using Perkin Elmer Operetta Automated Confocal microscope. Intensities were calculated by Harmony High-Content Imaging and Analysis Software.

### Biochemical assay for caspase activity in C2C12 cells

DEVD-AMC was used to measure caspase-3 like activity. Briefly, 20 μL of C2C12 cell extract was mixed with 185 μL of assay buffer containing HEPES 20 mM, 40 µM DEVD-AMC, and 1 mM DTT. Fluorescence was measured at 460-nm wavelength over 1 h. The activity was calculated as arbitrary fluorescence units min^−1^ mg^−1^ protein (AFU min^−1^ mg^−1^) and normalized to day 0 of a differentiation experiment.

### Cell-based assay for caspase activity in C2C12 cells

All siRNAs were reverse transfected in C2C12 cells in concentration of 100 nM and after 24 h cells were seeded at 50,000 cells/cm^2^ in 96-well plates. After 24 h, growth media was changed for differentiation media containing 5 µM IncuCyte® Caspase-3/7 Green Apoptosis Assay Reagent (Cat. No. 4440) and live cells imaging was performed every 2 h for 48 h using the IncuCyte® S3 Live-Cell Analysis System. The images were analysed with IncuCyte® Analysis Software. Cleavage of the substrate following caspase-3 activation leads to increased green fluorescence of the nuclei. The results are expressed as integrated green fluorescence intensity/mm^2^ normalized to the highest intensity at 48 h in cells transfected with scrambled siRNA.

### Cell death assay

Propidium iodide (PI; 0.5 µg mL^−1^) and Hoechst 33342 (1 µg mL^−1^) double staining was used to visualize dead cells and assess the extent of cell death. Under the conditions used here the PI positive cells were pyknotic, i.e. showed an apoptotic morphology and their appearance was blocked by caspase inhibitors. Cell death analyses were completed using Operetta high-content imaging system. The percentage of cell death was calculated as (number of PI positive cells/number of Hoechst positive cells) × 100.

### Assessment of the fusion index

Cells differentiated in µ-Plate 96-well plates (Ibidi GmbH) were washed to remove non-adherent cells and fixed in 4% paraformaldehyde at room temperature for 20 min. Cells were permeabilized (0.1% Triton X-100 for 15 min) and blocked with 10% FBS for 1 h before being stained with anti-Myosin Heavy Chain antibody (MF-20, Developmental Studies Hybridoma Bank, Iowa City, IA); 1:1000 in 10% FBS overnight and detected with a secondary goat anti-mouse antibody conjugated to Alexa Fluor 488 (Molecular Probes, Eugene, OR) 1:1000 for 1 h. Nuclei were counterstained with Hoechst 33342 (2 μg mL^−1^) for 1 min. Cells were visualized using a Perkin Elmer Operetta Automated Confocal microscope. The extent of fusion was quantified using the fusion index, defined as the percentage of nuclei found within multinucleate, myosin-positive cells.

### HEK293 cell culture, transfection, cell extract preparation, caspase assays and luciferase assays

DEVD-AMC was used to measure caspase-3 like activity and LEHD-pNA was used to measure caspase-9 activity. Briefly, 20 μL of cell extract was mixed with 185 μL of assay buffer containing HEPES 20 mM, 40 µM DEVD‐AMC or 200 μM LEHD-pNA, and 1 mM DTT. An increase in AMC fluorescence (ex 360 nm, em 460 nm) and an increase in pNA absorbance (400 nm) was measured for up to an hour. As extracts were diluted to the same protein concentration the activity was calculated as arbitrary fluorescence units min^−1^ (AFU min^−1^).

Split luciferase complementation assays for apoptosome formation were performed as previously described^19^. Briefly, ice cold extracts from N‐luc and C‐luc expressing cells (5 µL of each) were mixed in a well of a 96-well plate. Then the following reagents were added in order: cytochrome *c*, dATP (to 1.6 µM and 1 mM final concentration, respectively), and 30 µL of One-Glo luciferase assay system. The generation of light was then detected immediately using a Victor Plate reader at 25 °C over 15 min.

### Production of recombinant Apaf-1 using baculovirus expression system

Purified recombinant Apaf-1 (rApaf-1) was produced as previously described^19^: The cDNA encoding His-Tag Apaf-1 was cloned in pFastBac donor vector and transformed into DH10Bac *E. coli* competent cells. The recombinant bacmids were then extracted after antibiotic selection and blue-white screening and confirmed by partial sequencing. Sf21 insect cells were transfected with the Apaf-1 vector using Cellfectin reagent. The expression of proteins was analysed 3 days after transfection by immunoblotting. The viral stock was amplified to 70 mL and used for infection of 500 mL Sf21 cell culture at a density of 2.2 × 10^6^ cells mL^−1^. The cells were harvested 37 h post-infection. The cell pellet resuspended in 5 volumes of a buffer containing 20 mM HEPES pH 8.5, 10 mM KCl, 1.5 mM MgCl_2_, 1 mM DTT, 0.1 mM PMSF and Protease inhibitor cocktail and lysed by homogenization. S-100 extract was prepared and loaded on 3.5 mL nickel affinity column. The column washed using 10 volumes of washing buffer A (20 mM HEPES pH 8.5, 5 mM β-mercaptoethanol, 500 mM KCl, 20 mM imidazole and 10% glycerol), 2 volumes of washing buffer B (20 mM HEPES pH 8.5, 5 mM β-mercaptoethanol, 1 M KCl and 10% glycerol) and 2 volumes of washing buffer A. The column was eluted with 20 mM HEPES pH 8.5, 100 mM KCl, 250 mM imidazole, 5 mM β-mercaptoethanol and 10% glycerol. Buffer exchange was then performed in 20 mM HEPES pH 8.5, 10 mM KCl, 1 mM DTT and 10% glycerol using 100 molecular weight cut-off protein concentrators (Thermofisher, 88503). The proteins then aliquoted and stored at −80 °C.

### Thermal stability shift assay (TSA)

Human recombinant Apaf-1 was incubated either alone (monomeric rApaf-1) or with dATP (1 mM) and cytochrome *c* (1.6 µM) (oligomeric rApaf-1). Monomeric or oligomeric rApaf-1 was then incubated with DMSO (1.0%) or with M50054. Horse cytochrome *c* (1 mg mL^−1^) was incubated with DMSO (0.1%), M50054 or with ATP (10 mM). Samples were then heated for 7 min at a range of temperatures (42–90 °C) in a Veriti 96-well thermal cycler (Applied Biosystems, Foster City, CA) and then centrifuged at 14,000 rpm for 30 min to pellet insoluble protein. Ten microlitres of the supernatant was then subjected to SDS-PAGE. The amount of soluble protein was quantified by immunoblotting (rApaf-1) or by Coomassie blue staining (cytochrome *c*).

### Quantitation of protein levels after immunoblotting

The intensity of protein bands on immunoblots was quantified using Image J.

### Data analysis and statistics

For all numerical datasets with greater than two groups, one-way ANOVA was used to test for statistical significance within the dataset, and Tukey’s post hoc test was used to compute mean differences and assess statistical significance. All datasets with two or fewer groups were subject to Student’s unpaired *t*-test or a one sample *t*-test, respectively. Data analysis was performed using GraphPad Prism version 8 (GraphPad Software Inc, CA). Asterisks indicate significance at *P* < 0.05.

## Results

### Caspase-3 activity was in myoblasts that differentiated

**Fig. 1:**
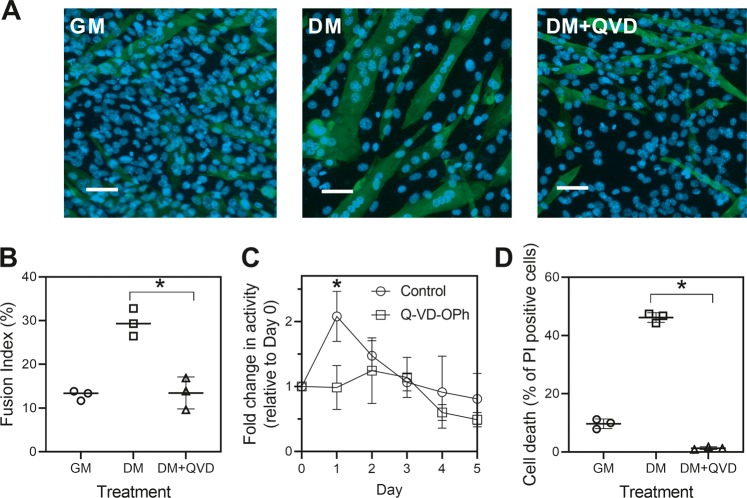
Caspase inhibitor blocks both cell death and differentiation in C2C12 cultures. **a** C2C12 cells in growth medium (GM) fused and expressed myosin heavy chain (green) when cultured in differentiation medium (DM) for 3 days. The caspase inhibitor Q-VD-OPh (DM + QVD) blocked cell fusion. Representative images are shown and scale bars represent 50 µm. **b** The effect of QVD on cell fusion was quantified. **c** Caspase-3 like activity was assessed in morphologically normal cells after apoptotic cells were washed away. **d** DM also induced caspase-dependent cell death. The data are the means ± SD of three independent experiments (*P < 0.05).

C2C12 myoblasts cultured in growth medium (GM) can be induced to differentiate by culturing the cells in differentiation medium (DM), which causes the cells to fuse into myotubes expressing myosin heavy chain (Fig. 1a). Caspase inhibitors, such as the pan-caspase inhibitor Q-VD-OPh, can prevent differentiation (Fig. 1a, DM + QVD and Fig. 1b)^8,10^. Culture conditions that induce C2C12 differentiation induce a transient increase in caspase-3 activity (Fig. 1c) and also induce significant levels of caspase-dependent apoptotic cell death (Fig. 1d). When measuring the caspase activity in differentiating cells (Fig. 1c), apoptotic cells were first washed away so the activity was associated with morphologically non-apoptotic cells. However, it was possible that early apoptotic cells were still present and responsible for the activity detected and that they would ultimately die and not directly participate in cell fusion.

**Fig. 2:**
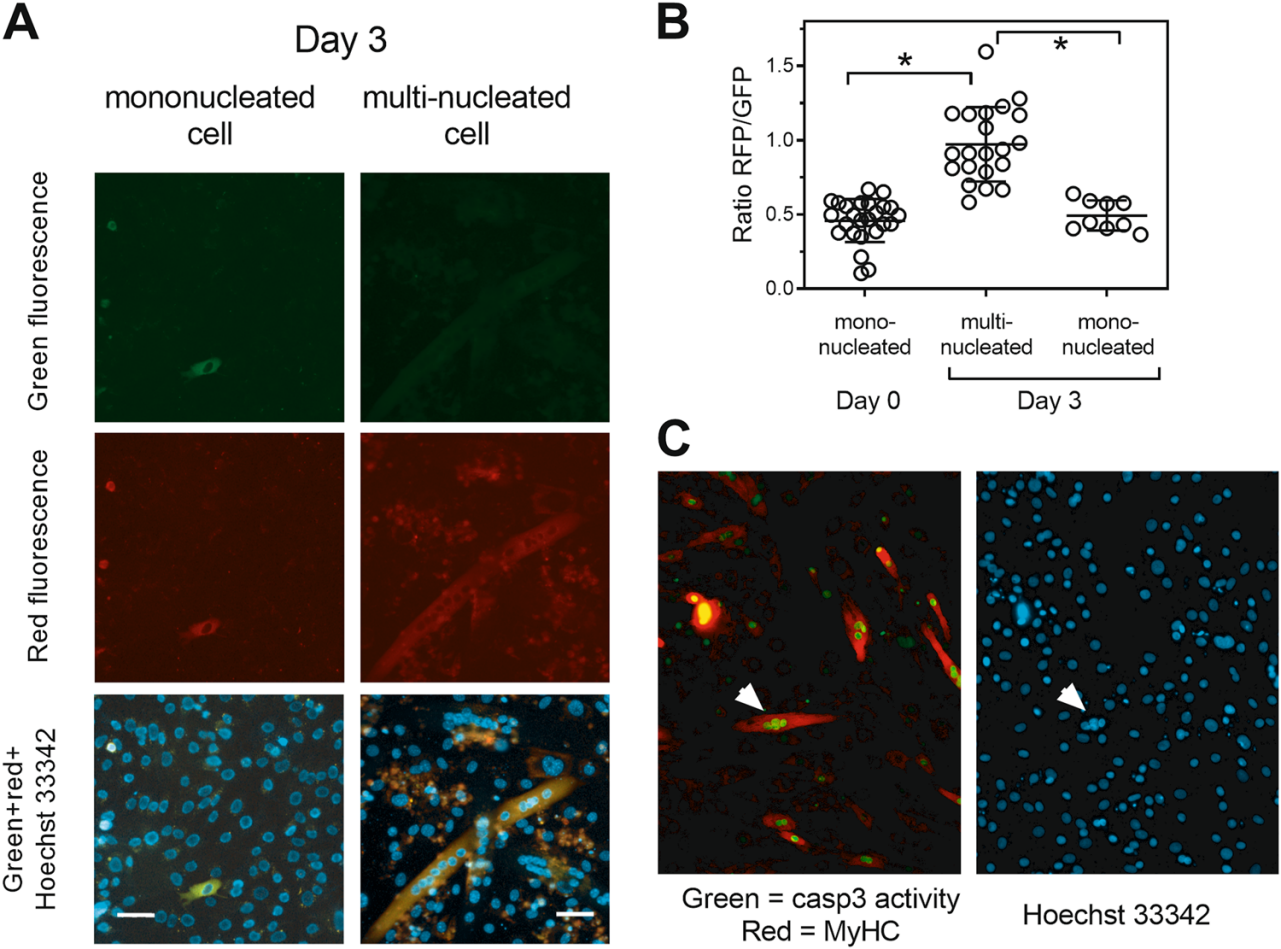
Caspase-3 is activated during differentiation. **a** Images of live mononucleated cells (left-hand panels) and multinucleated (right-hand panels) expressing the reporter on day 3 of differentiation. **b** Quantitation of the ratio of red to green fluorescence in mono and multinucleated cells on days 0 and 3. The data are the means ± SD of three independent experiments (*P < 0.05). **c** Caspase-3 activity detected using a cell permeable substrate is seen in multinucleated cells expressing MyHC (an example shown with white arrow). Scale bars represent 50 µm.

We therefore made use of the fluorescent protein exchange (FPX) biosensor for caspase-3 activity in cells^20^ to directly test whether cells that differentiated showed evidence of caspase activity. We first validated the usefulness of the biosensor, by inducing apoptosis in U2OS cells (Supplementary data). We next transfected C2C12 cells with the biosensor and induced differentiation. The transfection efficiency in C2C12 cells was relatively low (~20%) and only ~20% of cells differentiated, limiting the number of cells that both express the reporter and undergo differentiation. Nonetheless, after 3 days of differentiation, many red fluorescent multinucleated myotubes were observed (Fig. 2a). We next measured the ratio of red to green fluorescence in mononucleated and multinucleated myotubes at day 0 (before differentiation has begun) and on day 3 (when myotubes appear). The ratio of red to green fluorescence of myotubes was much increased compared with undifferentiated myoblasts on day 3 and myoblasts on day 0 (Fig. 2b). A two-plasmid reporter system gave similar results (Supplementary Figure 2). To corroborate these findings, we also used a cell permeable caspase substrate that results in green nuclear fluorescence when cleaved. These cells were fixed and stained to confirm expression of myosin heavy chain. On day 3 of differentiation multinucleated cells expressing myosin heavy chain with green nuclei were detected (Fig. 2c). Together, these data demonstrate that cells that activated caspase-3 fuse to form myotubes.

### Myoblast fusion required Apaf-1

**Fig. 3:**
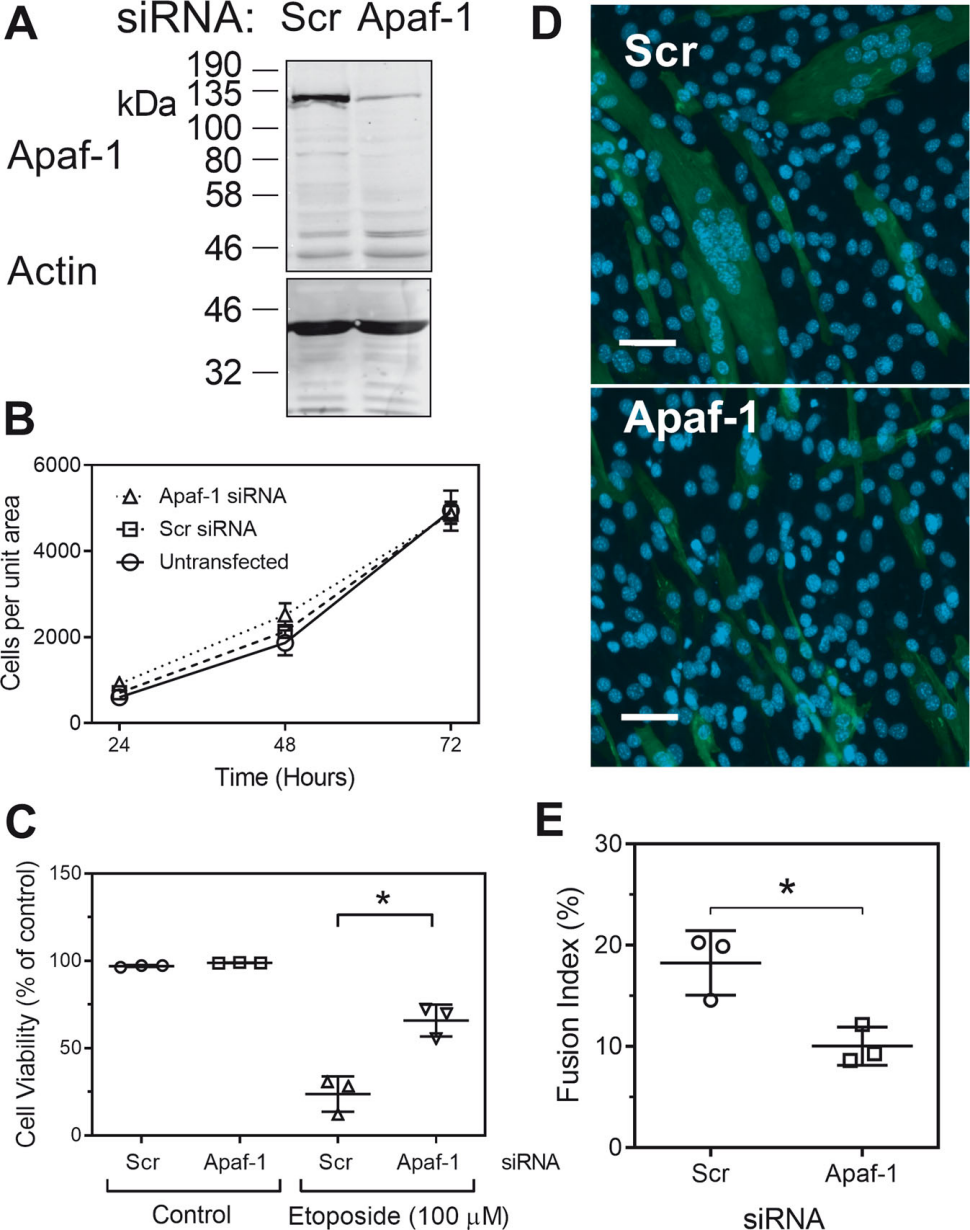
SiRNA against Apaf-1 blocks cell fusion. **a** Immunoblot showing extent of Apaf-1 knockdown by a scrambled control siRNA (Scr) and an Apaf-1 specific siRNA (Apaf-1). **b** The effect of siRNAs on cell survival and proliferation. **c** The effect of siRNAs on etoposide-induced cell death. **d** Representative images showing myosin heavy chain immunofluorescence and the effect of siRNA on cell fusion by day 3. Scale bars represent 50 µm. **e** Quantitation of inhibition of cell fusion by siRNAs by day 3. The data are the means ± SD of three independent experiments (*P < 0.05).

Previous reports show that overexpression of Bcl-XL and expression of a dominant negative caspase-9 can block myoblast differentiation and implicate the mitochondrial pathway of caspase activation in this process^8^. However, the role in differentiation of the caspase-9 activator Apaf-1 had not been tested. Therefore, we used specific siRNAs and knocked down Apaf-1 in C2C12 cells (Fig. 3a). Knockdown did not have any detectable effect on cell number (Fig. 3b), suggesting that Apaf-1 was not necessary to maintain C2C12 cell viability in either growth medium or differentiating medium. We also confirmed that the knockdown reduced etoposide-induced apoptosis as this is known to be Apaf-1 dependent and found that cell death was significantly reduced (Fig. 3c). Knockdown of Apaf-1 also markedly decreased cell fusion compared with transfection with a control (scrambled siRNA) (Fig. 3d and e). These data are consistent with the reported involvement of caspase-9 in myoblast differentiation^8^ and also suggest that the differentiation might be dependent on cytochrome *c*.

### Myoblast fusion required cytochrome *c*

**Fig. 4:**
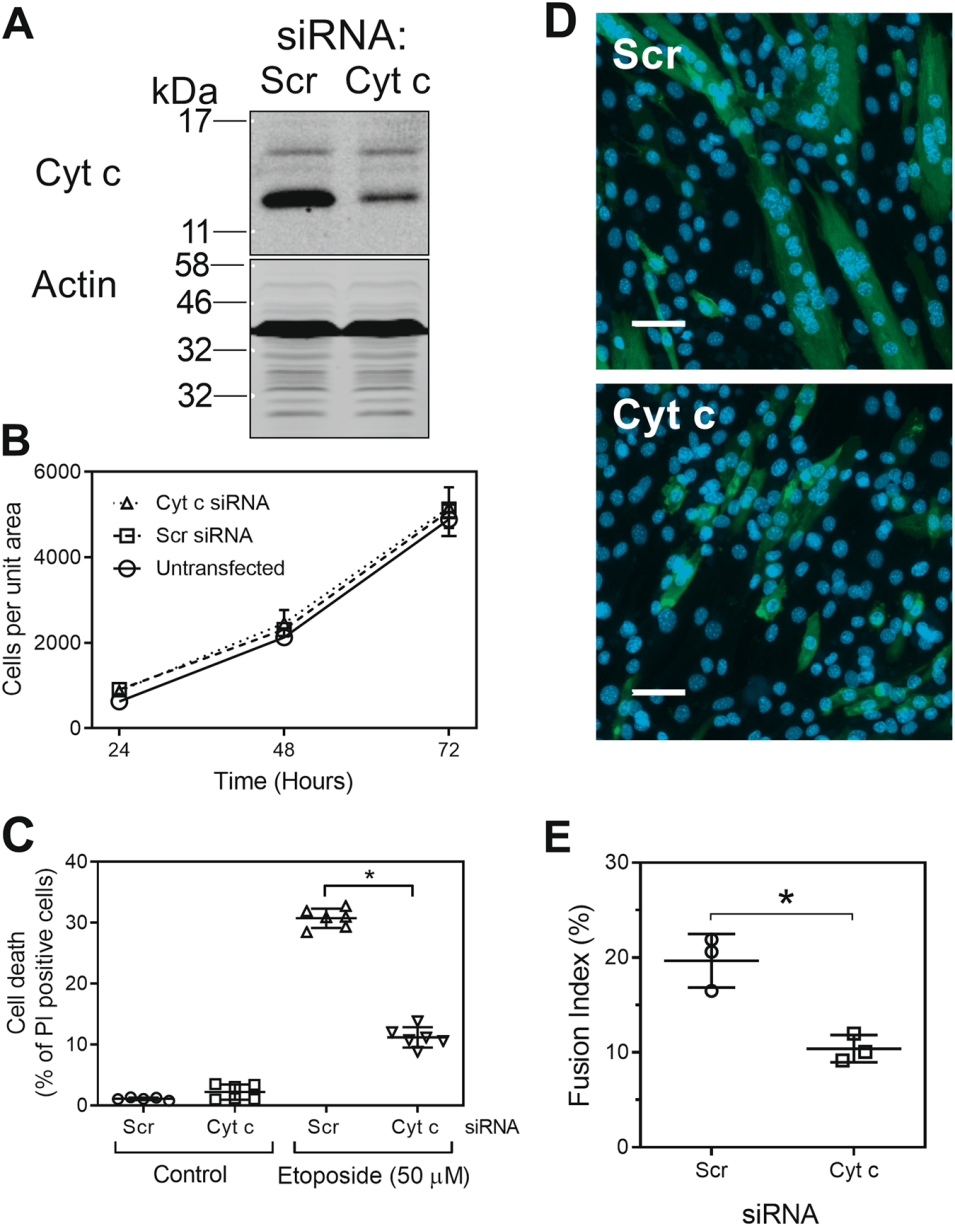
SiRNA against cytochrome *c* blocks cell fusion. **a** Immunoblot showing extent of Cyt *c* knockdown by a scrambled control siRNA (Scr) and a cytochrome *c* specific siRNA (Cyt *c*). **b** The effect of siRNAs on cell survival and proliferation. **c** The effect of siRNAs on etoposide-induced cell death. **d** Representative images showing myosin heavy chain immunofluorescence and the effect of siRNA on cell fusion by day 3. Scale bars represent 50 µm. **e** Quantitation of inhibition of cell fusion by siRNAs by day 3. The data are the mean ± SD of three independent experiments (*P < 0.05).

To address this question, cytochrome *c* was knocked down in C2C12 cells using specific siRNAs and the effect on cell differentiation assessed. Cytochrome *c* expression could be significantly reduced (Fig. 4a) without any detectable effect on cell number (Fig. 4b), suggesting that cytochrome *c* was not necessary to maintain C2C12 cell viability in either growth medium or differentiating medium. We also confirmed that knockdown of cytochrome *c* reduced etoposide-induced cell death (Fig. 4c). Cell fusion was also markedly decreased when cells were transfected with siRNA against cytochrome *c*, compared with transfection with a control (scrambled siRNA) (Fig. 4d and e). These data show that cytochrome *c* was required for differentiation, and together with data showing a dependence on Apaf-1, a role for caspase-9 and sensitivity to Bcl-XL overexpression strongly argue for the importance of the mitochondrial pathway for caspase activation in myoblast differentiation.

However, Apaf-1 has a role in controlling cell-cycle checkpoints besides its role in the activation of caspases^21^. At the same time, knocking down cytochrome *c* could have other effects besides compromising apoptosome formation. We therefore wanted to test the idea that the mitochondrial pathway was involved using a different but complementary approach. To do this, we used our discovery that M50054, a small molecule inhibitor of caspase activation acts by binding to activated Apaf-1.

### M50054 inhibited active Apaf-1

**Fig. 5:**
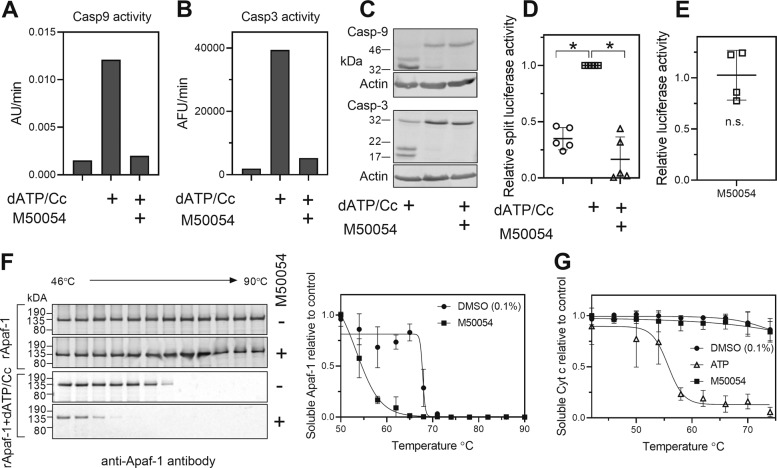
M50054 inhibits active Apaf-1. The effect of M50054 on caspase-9 (**a**) and -3 (**b**) activity induced by 1 mM dATP and 1.6 µM cytochrome *c* (dATP/Cc). After 15 min incubation with dATP/Cc the activity of caspase-9 like activity was measured with LEHD-pNA and the activity of caspase-3 activity measured with DEVD-AFC. **c** Immunoblotting to assess the effect of M50054 on dATP and cytochrome *c* induced processing of caspase-9 and -3. The results of (**a**), (**b**) and (**c**) are from one cell extract and are typical of three independent experiments. **d** The effect of M50054 (500 µM) on Apaf-1:Apaf-1 interactions were assessed using a split luciferase complementation assay. **e** The effect of M50054 (500 µM) on luciferase activity relative to DMSO (0.1% v/v). **f** The binding of M50054 to inactive and active Apaf-1 was assessed using a thermal stability assay. **g** The binding of M50054 to cytochrome *c* was assessed using a thermal stability assay. 10 mM ATP was used as a positive control. The data are the mean ± SD of three independent experiments (*P < 0.05).

M50054 is a commercially available chemical that prevents caspase activation by a previously unknown mechanism^22^. Using extracts from HEK293 cells, we activated caspase-9 and -3 by addition of dATP and cytochrome *c* (dATP/Cc) and confirmed that the activity of both enzymes was markedly decreased (Fig. 5a and b). We also investigated the processing of caspases-3 and -9 and found that in both cases processing was prevented (Fig. 5c). These data are consistent with previous reports that M50054 blocks caspase activation and suggest that M50054 acts upstream of caspase-9. We next used a split luciferase complementation assay to test if M50054 inhibited formation of the apoptosome^19^. In this assay, cell extracts from cells expressing Apaf-1 fused to an N-terminal fragment of luciferase and extracts from cells expressing Apaf-1 fused to a C-terminal fragment of luciferase were mixed. dATP and cytochrome *c* was then added and the interaction of Apaf-1 molecules in the apoptosome was detected as an increase in luciferase activity. M50054 inhibited dATP/Cc-induced luciferase activity (Fig. 5d), but it did not directly inhibit luciferase activity (Fig. 5e), which suggested that M50054 acted to prevent apoptosome formation. But M50054 could have been acting indirectly by binding to one of the many proteins in the extract. We therefore used purified recombinant Apaf-1 in a thermal stability assay^23^ to test whether M50054 directly interacted with Apaf-1 (Fig. 5f). Inactive Apaf-1 (in the absence of dATP/Cc) was a very thermal stable protein, and M50054 did not alter the thermal stability, suggesting that M50054 cannot bind to Apaf-1 in its monomeric (inactive) state. We next tested Apaf-1 that was activated by pre-incubation with dATP/Cc. Activated Apaf-1 was less thermal stable than the inactive protein, probably reflecting the conformational changes associated with activation. Moreover, addition of M50054 further altered the thermal stability of Apaf-1, suggesting that M50054 was binding either to Apaf-1 or to cytochrome *c*. We therefore tested if M50054 could bind cytochrome *c* using a thermal stability assay (Fig. 5g). We used ATP as a positive control as this is known to bind cytochrome *c* and inhibit apoptosome formation^24,25^. While ATP dramatically altered the thermal stability of cytochrome *c*, M50054 could not. We therefore concluded that M50054 acted to block caspase activation by binding to activated Apaf-1.

### M50054 blocked Apaf-1-dependent cell death and myoblast fusion

**Fig. 6:**
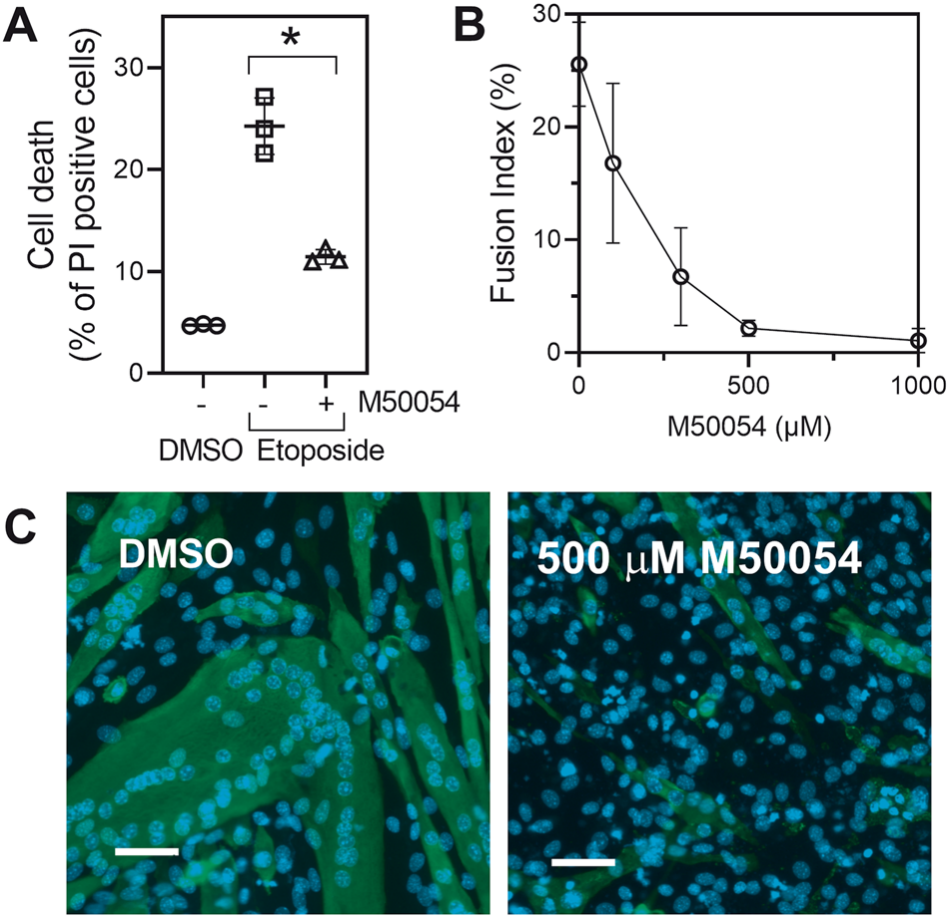
M50054 blocked cell fusion. **a** The effect of M50054 (500 µM) on etoposide (50 µM)-induced cell death was assessed. **b**, **c** The effect of M50054 on cell fusion was assessed. The data are the mean ± SD of three independent experiments (*P < 0.05). Scale bars represent 50 µm.

We next tested whether Apaf-1-dependent apoptosis or cell fusion were blocked by M50054. Etoposide-induced cell death in C2C12 myoblasts is reduced by Apaf-1- and cytochrome *c-*specific siRNAs (Figs. 3c and 4c). We therefore treated C2C12 cells with etoposide in the presence or absence of M50054 and found that M50054 was an effective inhibitor of cell death (Fig. 6a). We then went on to investigate whether M50054 inhibited cell differentiation by assessing the effect of M50054 on cell fusion (Fig. 6b and c). M50054 showed a concentration-dependent inhibition of cell fusion with the IC50 value (~200 µM) similar to that reported for inhibition of caspase activation and cell death (~300 µM)^22^. These data suggest that cell fusion required the activation of Apaf-1 by cytochrome *c*. Considering the experiments using specific siRNAs against Apaf-1 and cytochrome *c*, and our previous report that fusion requires caspase-9 activation^8^, it is clear that caspase-3 activation in differentiating C2C12 cells is activated by the mitochondrial or intrinsic pathway, even though the cells do not die.

### Myoblast fusion required Bid

**Fig. 7:**
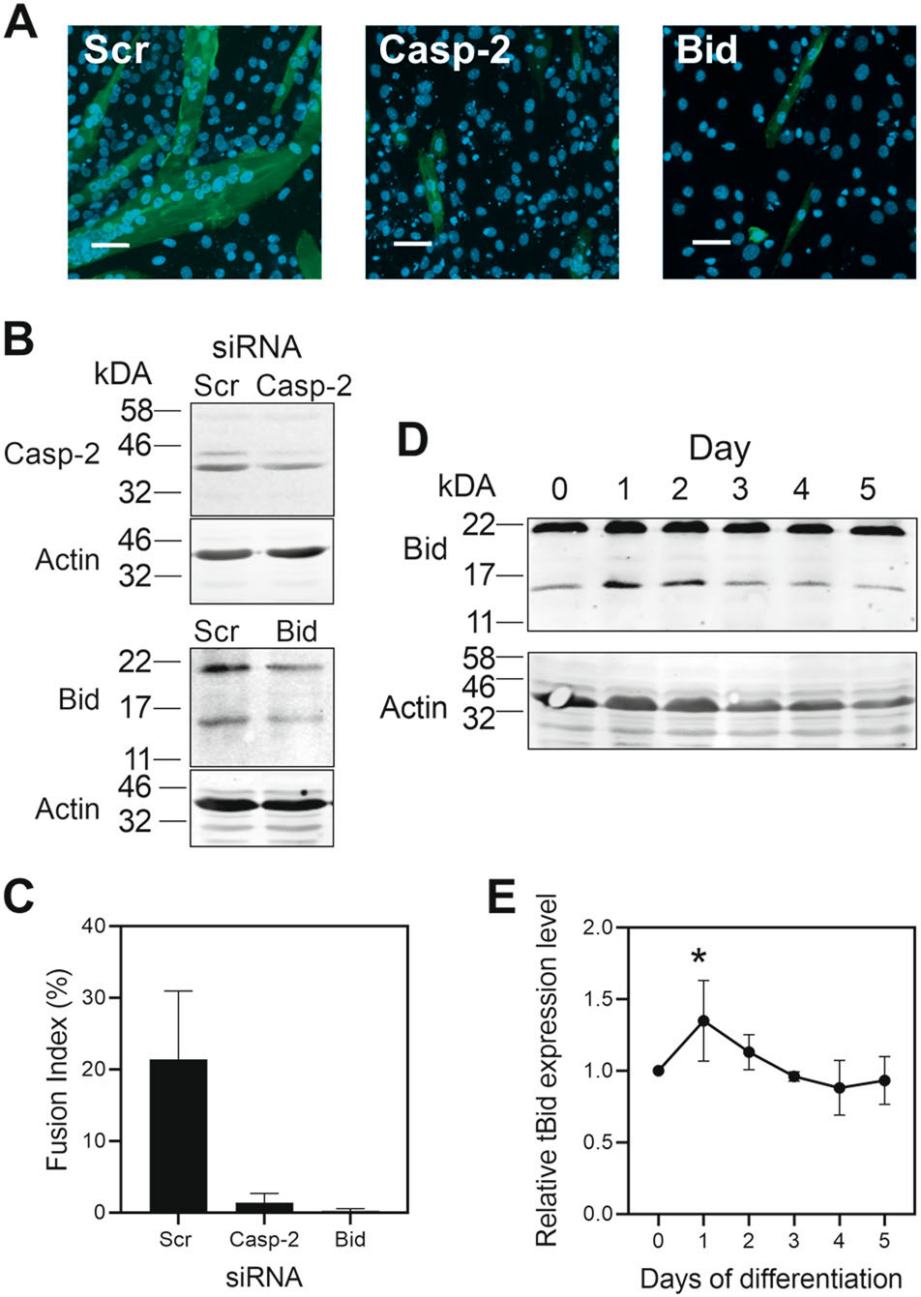
SiRNA against Bid blocked cell fusion. C2C12 cells were transfected with siRNAs and differentiation induced. After 3 days in differentiation medium the effect on cell fusion was assessed. **a** Immunofluorescence showing the effect of specific siRNAs on cell fusion compared with that of a scrambled siRNA. **b** Knockdown of caspase-2 and Bid assessed by immunoblotting. **c** Quantitation of the effect of siRNAs on cell fusion. **d** tBid levels during differentiation assessed by immunoblotting and quantified (**e**). Data are the mean ± SD of three independent experiments (*P < 0.05). Scale bars represent 50 µm.

Caspase-2 has been previously shown to play a role in C2C12 cell differentiation^9^, so we confirmed that caspase-2 was required for differentiation by using an RNAi approach (Fig. 7b) and assessing cell fusion (Fig. 7a and c). We found that caspase-2 siRNAs markedly decreased fusion, and because caspase-2 can cleave Bid^26^ to tBid and trigger cytochrome *c* release in apoptosis^27,28^, we tested whether Bid also played a role in C2C12 differentiation. We found that siRNAs specific for Bid (Fig. 7b) markedly inhibited fusion (Fig. 7a and c). We also detected the transient appearance of tBid on day 1 of differentiation (Fig. 7d and e), which is co-incident with the peak caspase activity during differentiation (Fig. 1a).

### Knockdown of caspase-2 or Bid have different effects from knockdown of Apaf-1 or cytochrome *c*

**Fig. 8:**
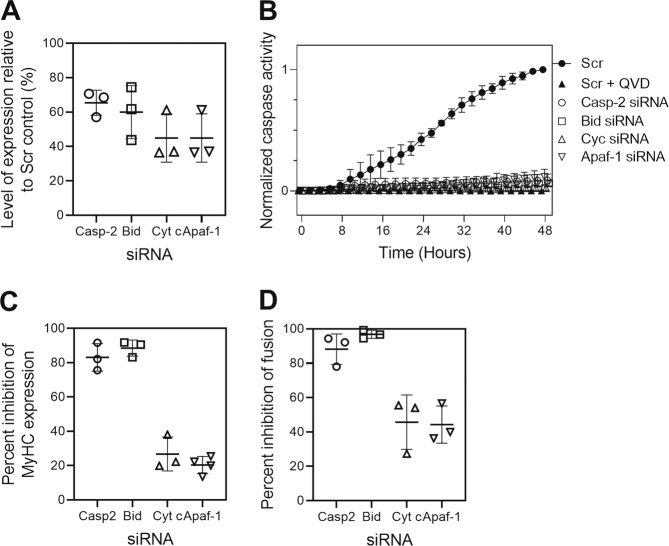
The effects of Casp2 and Bid siRNA compared with Cyt *c* and Apaf-1 siRNA. **a** The protein levels after siRNA treatment relative to the effect of a scrambled siRNA. Quantitation performed using fluorescent secondary antibodies and Licor™ scanner. **b** Live-cell imaging of caspase-3 activity using the Incucyte. C2C12 cells transfected with different siRNAs (100 nM) were incubated with a cell permeable substrate (5 µM) and images were captured every 2 h for 48 h. The data were normalized to the maximum fluorescence intensity of the control siRNA at 48 h and expressed as the mean ± SD of three independent experiments (*P < 0.05). Percent inhibition of myosin heavy chain expression (**c**) and cell fusion (**d**) by the different siRNAs. The data are the mean ± SD of three independent experiments.

The specific siRNAs against caspase-2, Bid, Apaf-1 and cytochrome *c* reduced expression of their specific targets by similar amounts (Fig. 8a). All four specific siRNAs also reduced caspase-3-like activity similarly (Fig. 8b). However, the effects of the siRNAs on two indicators of differentiation (myosin heavy chain expression and cell fusion) were significantly different. Knocking down Apaf-1 or cytochrome *c* inhibited expression of myosin heavy chain by ~20% (Fig. 8c and Supplementary Figure 3) and cell fusion by ~40% (Fig. 8d). In contrast, knocking down caspase-2 or Bid inhibited expression levels of myosin heavy chain by ~80% (Fig. 8c) and cell fusion by ~90% (Fig. 8d). These data show that interfering upstream of mitochondria by reducing the levels of caspase-2 or Bid had a more profound effect on differentiation than interfering at or downstream of apoptosome formation, even though caspase-3 activity was similarly affected. These data suggest that caspase-2 and Bid play additional roles controlling myogenesis besides activation of the mitochondrial pathway.

We next tested whether overexpression of caspase-2, Bid or Apaf-1 was sufficient to induce differentiation. To do this we compared the effect of overexpressed caspase-2, dominant negative caspase-2, Bid, an uncleavable Bid, tBid and Apaf-1 on C2C12 differentiation. While overexpression of Bid, tBid and Apaf-1 caused an increase in cell death, no increase in cell fusion was detected following overexpression of any of the genes (Supplementary Figure 4), suggesting that overexpression was not sufficient for differentiation.

## Discussion

The idea that killer caspases can induce cell fates other than cell death has now been established using several different in vitro and in vivo model systems (reviewed in refs. ^2,29,30^). In some models it appears that the killer caspases act indirectly: the caspase activity kills the cell and the dead or dying cells provides a signal to surrounding stem or progenitor cells to drive differentiation as part of a repair and regeneration process^31^. In other cases, the caspase activity is in the cell that goes on to differentiate^3^. In muscle it was unclear which of these mechanisms was occurring. Hochreiter-Hufford et al.^18^ reported that dead and dying myoblasts present phosphatidylserine (PS) on their surface and that this PS is a signal to drive differentiation. We have failed to reproduce these data (data not shown). However, we were able to detect evidence of caspase activation inside cells that have differentiated using different reporters of caspase activity in live cells. This is the first evidence showing a cell autonomous role for killer caspases in myoblast differentiation.

We had previously shown caspase-9, which is most clearly associated with the intrinsic or mitochondrial apoptotic pathway, was involved in myoblast differentiation^8^. During cell death, cell-stress causes mitochondria to release cytochrome *c*. Once in the cytosol, cytochrome *c* binds to Apaf-1, an event that triggers Apaf-1 oligomerization into a heptameric complex (the apoptosome). The apoptosome activates caspase-9, which in turn activates caspase-3. Active caspase-3 then cleaves a range of substrates and kills the cell^32^. Here we have shown that differentiation in mouse myoblasts required activated Apaf-1 and cytochrome *c*. This differentiation was also dependent on Bid expression and associated with Bid cleavage to tBid, a protein that releases cytochrome *c* from mitochondria.

However, Apaf-1, cytochrome *c* and Bid all have reported non-apoptotic functions. For example, an Apaf-1 isoform that cannot bind cytochrome *c* or activate caspases can control a cell-cycle checkpoint^21^, cytochrome *c* is required for electron transport in the mitochondria and Bid is involved in DNA-damage responses when phosphorylated by ATM^33^. Thus, while reducing expression of these proteins compromised differentiation, it was possible that this was because several different and unrelated processes besides the mitochondrial death pathway were being affected. To address this issue, we took advantage of our observation that M50054 blocked caspase-9 activation by binding to Apaf-1 that had been activated by cytochrome *c*, but not to inactive Apaf-1. As expected, M50054 blocked cell death induced by the mitochondrial pathway. It also blocked the differentiation of myoblasts. This pharmacological approach complemented the RNAi experiments and together our data strongly support the idea that the mitochondrial death pathway is capable of driving cell fusion as well as cell death.

This is the first report of a small molecule that binds to active Apaf-1 and prevents caspase activation. Other Apaf-1 inhibitors interfere with the cell-cycle checkpoint function^34^, which does not require the apoptosome^21^. In the case of another inhibitor (NS3694), it is not clear that the inhibitor even targets Apaf-1^35^. M50054 is therefore a valuable pharmacological tool for complementing genetic studies and demonstrating apoptosome involvement. Indeed, the effect of M50054 on regeneration in frog^36^ and fish models^37^ suggests that these are apoptosome-dependent processes.

This is also the first report to integrate caspase-2 with downstream events that control cell fusion by showing a role for Bid. We observed an interesting difference between the effects of targeting genes upstream of mitochondrial events and targeting events downstream of mitochondria. While all siRNAs had similar degrees of knockdown for their specific targets, and also similar effect on caspase-3 activity, siRNA against caspase-2 and Bid affected both cell fusion and expression of myosin heavy chain, while siRNA against Apaf-1 and cytochrome *c* affected cell fusion and myosin heavy chain expression to a lesser degree. The difference between the effects of caspase-2 and Bid siRNAs versus Apaf-1 and cytochrome *c* siRNAs suggests that caspase-2 and Bid control redundant pathways leading to differentiation. Thus, caspase-2 and Bid lie upstream of an apoptosome-dependent pathway but also control an apoptosome-independent pathways. Therefore, our data also reveal a hierarchy of caspases, with caspase-2 playing a more important role than caspase-3.

Because muscle has high demands for energy, mitochondrial function is intimately linked to myogenesis^38,39^, with key roles for mitochondrial reactive oxygen species, biogenesis, fission and fusion being described^38,40–43^. Experimental data further illustrate the importance of mitochondrial function for proper myogenesis^44^ as do the range of mitochondrial myopathies^45–47^. Interestingly, one myopathy, Infantile-onset multisystem disease with progressive muscle weakness is caused by mutations in Bit-1/PTRH-2, which controls Bcl-2 expression^48,49^. Myoblasts from PTRH-2 null mice show dysregulated caspase activity and defective cell fusion^50^, potentially linking the apoptosome-dependent cell fusion shown here to human disease.

In conclusion, we have answered two important questions regarding the caspase-dependent differentiation of myoblasts. Firstly, we have shown that the caspase activity is in the cells that differentiate. So, while dead cells may also signal to enhance differentiation, this is not the only caspase-dependent mechanism in play. Secondly, we have provided genetic and pharmacological evidence that the apoptosome is required for differentiation. Together with reports that differentiation requires caspase-9 activity and is blocked by Bcl-XL overexpression, these data strongly support the idea that the mitochondrial death pathway is not just a death pathway, but also plays a role in inducing non-cell death fates. In addition, we have also provided evidence that caspase-2 plays a role in differentiation upstream of caspase-9, and that caspase-2 and Bid play additional regulatory roles during myogenesis.

## Supplementary information

Supplementary data legends & tables

Supplementary figure 1.

Supplementary figure 2.

Supplementary Figure 3

Supplementary Figure 4

## References

[1] Parrish AB, Freel CD, Kornbluth S (2013). Activation and function. Cold Spring Harb. Perspect. Biol..

[2] Aram L, Yacobi-Sharon K, Arama E (2017). CDPs: caspase-dependent non-lethal cellular processes. Cell Death Differ..

[3] Arama E, Agapite J, Steller H (2003). Caspase activity and a specific cytochrome C are required for sperm differentiation in *Drosophila*. Dev. Cell.

[4] Black S (2004). Syncytial fusion of human trophoblast depends on caspase 8. Cell Death Differ..

[5] Campbell DS, Holt CE (2003). Apoptotic pathway and MAPKs differentially regulate chemotropic responses of retinal growth cones. Neuron.

[6] Fujita J (2008). Caspase activity mediates the differentiation of embryonic stem cells. Cell Stem Cell.

[7] Mogi M, Togari A (2003). Activation of caspases is required for osteoblastic differentiation. J. Biol. Chem..

[8] Murray TVA (2008). A non-apoptotic role for caspase-9 in muscle differentiation. J. Cell Sci..

[9] Boonstra K, Bloemberg D, Quadrilatero J (2018). Caspase-2 is required for skeletal muscle differentiation and myogenesis. Biochim. Biophys. Acta - Mol. Cell Res..

[10] Fernando P, Kelly JF, Balazsi K, Slack RS, Megeney LA (2002). Caspase 3 activity is required for skeletal muscle differentiation. Proc. Natl Acad. Sci. USA.

[11] Gyrd-Hansen M (2006). Apoptosome-independent activation of the lysosomal cell death pathway by caspase-9. Mol. Cell. Biol..

[12] Bitzer M (2002). Caspase-8 and Apaf-1-independent caspase-9 activation in Sendai virus-infected cells. J. Biol. Chem..

[13] Bloemberg D, Quadrilatero J (2014). Mitochondrial pro-apoptotic indices do not precede the transient caspase activation associated with myogenesis. Biochim. Biophys. Acta - Mol. Cell Res..

[14] Li F (2010). Apoptotic cells activate the ‘phoenix rising’ pathway to promote wound healing and tissue regeneration. Sci. Signal.

[15] Ryoo HD, Gorenc T, Steller H (2004). Apoptotic cells can induce compensatory cell proliferation through the JNK and the wingless signaling pathways. Dev. Cell.

[16] Huh JunR, Ming Gou BAH (2004). Compensatory proliferation induced by cell death in the *Drosophila* wing disc requires activity of the apical cell death caspase DRONC in a non-apoptotic role. Curr. Biol..

[17] Boland K, Flanagan L, Prehn JHM (2013). Paracrine control of tissue regeneration and cell proliferation by Caspase-3. Cell Death Dis..

[18] Hochreiter-Hufford AE (2013). Phosphatidylserine receptor BAI1 and apoptotic cells as new promoters of myoblast fusion. Nature.

[19] Tashakor A (2019). A new split-luciferase complementation assay identifies pentachlorophenol as an inhibitor of apoptosome formation. FEBS Open Bio..

[20] Ding Y (2015). Ratiometric biosensors based on dimerization-dependent fluorescent protein exchange. Nat. Methods.

[21] Zermati Y (2007). Nonapoptotic role for Apaf-1 in the DNA damage checkpoint. Mol. Cell.

[22] Tsuda T (2001). Inhibitory effect of M50054, a novel inhibitor of apoptosis, on anti-Fas-antibody-induced hepatitis and chemotherapy-induced alopecia. Eur. J. Pharmacol..

[23] Molina DM, Jafari R, Ignatushchenko M, Seki TD (2013). Monitoring drug target engagement in cells and tissues using the cellular thermal shift assay. Science.

[24] Chandra D (2006). Intracellular nucleotides act as critical prosurvival factors by binding to cytochrome C and inhibiting apoptosome. Cell.

[25] Samali A (2007). Identification of an inhibitor of caspase activation from heart extracts; ATP blocks apoptosome formation. Apoptosis.

[26] Guo Y, Srinivasula SM, Druilhe A, Fernandes-Alnemri T, Alnemri ES (2002). Caspase-2 induces apoptosis by releasing proapoptotic proteins from mitochondria. J. Biol. Chem..

[27] Gross A (1999). Caspase cleaved BID targets mitochondria and is required for cytochrome c release, while BCL-X(L) prevents this release but not tumor necrosis factor-R1/Fas death. J. Biol. Chem..

[28] Li H, Zhu H, Xu CJ, Yuan J (1998). Cleavage of BID by caspase 8 mediates the mitochondrial damage in the Fas pathway of apoptosis. Cell.

[29] White K, Arama E, Hardwick JM (2017). Controlling caspase activity in life and death. PLoS Genet..

[30] Connolly PF, Jäger R, Fearnhead HO (2014). New roles for old enzymes: Killer caspases as the engine of cell behavior changes. Front. Physiol..

[31] Fogarty CE, Bergmann A (2017). Killers creating new life: caspases drive apoptosis-induced proliferation in tissue repair and disease. Cell Death Differ..

[32] Cain K, Bratton SB, Cohen GM (2002). The Apaf-1 apoptosome: a large caspase-activating complex. Biochimie.

[33] Kamer I (2005). Proapoptotic BID is an ATM effector in the DNA-damage response. Cell.

[34] Mondragón L (2009). A chemical inhibitor of Apaf-1 exerts mitochondrioprotective functions and interferes with the intra-S-phase DNA damage checkpoint. Apoptosis.

[35] Lademann U (2003). Diarylurea compounds inhibit caspase activation by preventing the formation of the active 700-kilodalton apoptosome complex. Mol. Cell. Biol..

[36] Kha CX, Tseng KAS (2018). Developmental dependence for functional eye regrowth in Xenopus laevis. Neural Regen. Res..

[37] Sîrbulescu RF, Zupanc GKH (2010). Inhibition of caspase-3-mediated apoptosis improves spinal cord repair in a regeneration-competent vertebrate system. Neuroscience.

[38] Rochard P (2000). Mitochondrial activity is involved in the regulation of myoblast differentiation through myogenin expression and activity of myogenic factors. J. Biol. Chem..

[39] Wagatsuma A, Sakuma K (2013). Mitochondria as a potential regulator of myogenesis.. Sci. World J..

[40] Sin J (2016). Mitophagy is required for mitochondrial biogenesis and myogenic differentiation of C2C12 myoblasts. Autophagy.

[41] Collu-Marchese M, Shuen M, Pauly M, Saleem A, Hood DA (2015). The regulation of mitochondrial transcription factor A (Tfam) expression during skeletal muscle cell differentiation. Biosci. Rep..

[42] Malinska D, Kudin AP, Bejtka M, Kunz WS (2012). Changes in mitochondrial reactive oxygen species synthesis during differentiation of skeletal muscle cells. Mitochondrion.

[43] Kim B (2013). Inhibition of Drp1-dependent mitochondrial division impairs myogenic differentiation. Am. J. Physiol. - Regul. Integr. Comp. Physiol.

[44] Hong J (2014). Mitochondrial complex I deficiency enhances skeletal myogenesis but impairs insulin signaling through SIRT1 inactivation. J. Biol. Chem..

[45] DiMauro S (2006). Mitochondrial myopathies. Curr. Opin. Rheumatol..

[46] Falk MJ, Sondheimer N (2010). Mitochondrial genetic diseases. Curr. Opin. Pediatr..

[47] Bloemberg D, Quadrilatero J (2016). Effect of mitochondrial fission inhibition on C2C12 differentiation. Data Br..

[48] Doe J (2017). PTRH2 gene mutation causes progressive congenital skeletal muscle pathology. Hum. Mol. Genet..

[49] Hu H (2014). Mutations in PTRH2 cause novel infantile-onset multisystem disease with intellectual disability, microcephaly, progressive ataxia, and muscle weakness. Ann. Clin. Transl. Neurol..

[50] Griffiths GS (2015). Bit-1 is an essential regulator of myogenic differentiation. J. Cell Sci..

