# Supplementary material for: Apoptosome-dependent myotube formation involves activation of caspase-3 in differentiating myoblasts
Source: Cell Death Dis. 2020 May 4;11(5):308. doi: 10.1038/s41419-020-2502-4 (PMC7198528; doi:10.1038/s41419-020-2502-4)
Supplement: Supplementary file 1 — Supplementary data legends & tables [file 41419_2020_2502_MOESM1_ESM.docx]

**Supplementary data**

**Supplementary Figure S1. Reporter validation.**

(A) Cartoon showing the caspase-3 biosensor and increased red fluorescence after caspase-3 cleavage. (B) Fluorescence microscopy of control (0.1%DMSO), Etoposide (50 µM) and Etoposide (50 µM) plus Q-VD-OPh treated cells at 24 hours. Inserts show examples of normal and apoptotic morphology. (C) Etoposide induced cell death was blocked by Q-VD-OPh, and apoptotic cells showed increased ratio of red to green fluorescence compared to morphologically normal cells.

**Supplementary Figure S2. Alternative reporter using two plasmids.**

A two-plasmid reporter system (A) was transiently expressed in C2C12 cells. Caspase activity in live cells was assessed after three days of differentiation (B). The ratio of RFP to GFP fluorescence of nuclei in mono-nucleated or multi-nucleated cells was then quantified (C). Transient transfection of the two plasmids needed led to fewer cells reporting caspase activity compared to the reporter that uses a single plasmid (Figure 2). Therefore, while both reporter systems gave the same result, the one plasmid reporter system gave clearer data.

**Supplementary Figure 3. Effect of siRNAs on MyHC expression**

The expression of MyHC was quantified by assessing the pixel area staining with an anti-MyHC antibody. (A) Effect of caspase-2 and Bid siRNA on MyHC expression compared to a scrambled siRNA (Scr). (B) Effect of cytochrome *c* siRNA on MyHc expression. (C) Effect of Apaf-1 siRNA on MyHc expression.

**Supplementary Figure 4. The effect of overexpression on cell number and cell fusion**

Caspase-2, dominant negative caspase-2, Bid, uncleavable Bid, tBid and Apaf-1 where overexpressed in C2C12 cells cultured in growth medium. The effect on cell death was assessed after 24 hours from nucleofection and the effect on cell fusion assessed after 72 hours from culturing remaining cells in growth medium. Data are the mean ± SD of three independent experiments (*, P < 0.05).

**Supplementary Table 1: Details of primary antibodies used in this research**

| **Primary Antibodies** | **Company** | **Catalogue No** |
| --- | --- | --- |
| Myosin 4 Monoclonal Antibody(MF20) | Thermo Fisher | 14-6503-82 |
| Cytochrome c | BD Pharmingen | 556433 |
| Apaf1 monoclonal antibody | AdipoGen, | AG-20T-0134-c100 |
| Caspase-2 (C2) Mouse mAb | Cell Signaling | 2224S |
| BID Antibody (Mouse Specific) | Cell Signaling | 2003 |
| Human/Mouse BID Antibody | R&D System | MAB860-SP |
| Beta Actin Antibody | Proteintech | 66009-1-Ig |

Supplementary Table 2: Details of secondary antibodies used in this research

| **Secondary Antibodies** | **Company** | **Catalogue No** |
| --- | --- | --- |
| Alexa Fluor 488 (Goat anti-Mouse IgG Secondary Antibody) | Invitrogen | A-11029 |
| Alexa Fluor 647(Goat anti-Rabbit IgG (H+L)) | Invitrogen | A-21245 |
| Goat anti-rat | Li-cor Biosciences | 925-32219 |
| Goat anti-rabbit | Li-cor Biosciences | 926-32211 |
| Goat anti mouse | Li-cor Biosciences | 926-68020 |

Supplementary Table 3. Details of siRNAs used in this research

| **siRNA** | **Company** | **Catalogue No** |
| --- | --- | --- |
| Apaf1 mouse siRNA oligo duplex | Origene | SR422513 |
| Cytochrome c siRNA | Origene | SR401266 |
| Caspase-2 siRNA, MISSION® esiRNA | Sigma | EMU017971 |
| Bid siRNA, MISSION® esiRNA | Sigma | EMU087401 |
| Apaf1 siRNA, MISSION® esiRNA | Sigma | EMU084081 |
