# Supplementary figures and images for: Apoptosome-dependent myotube formation involves activation of caspase-3 in differentiating myoblasts
Source: Cell Death Dis. 2020 May 4;11(5):308. doi: 10.1038/s41419-020-2502-4 (PMC7198528; doi:10.1038/s41419-020-2502-4)

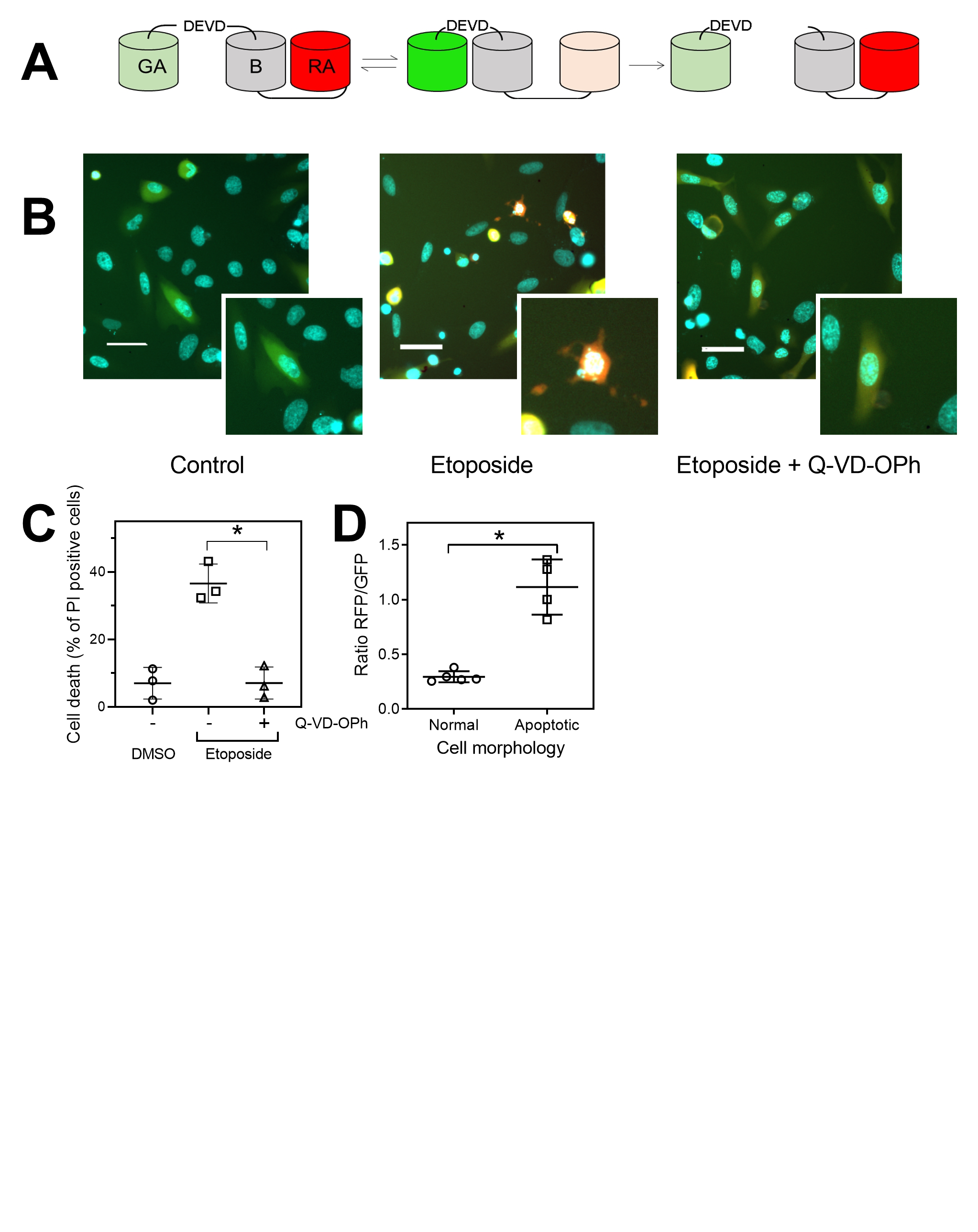

Supplement: Supplementary file 2 — Supplementary figure 1. [file 41419_2020_2502_MOESM2_ESM.tif]

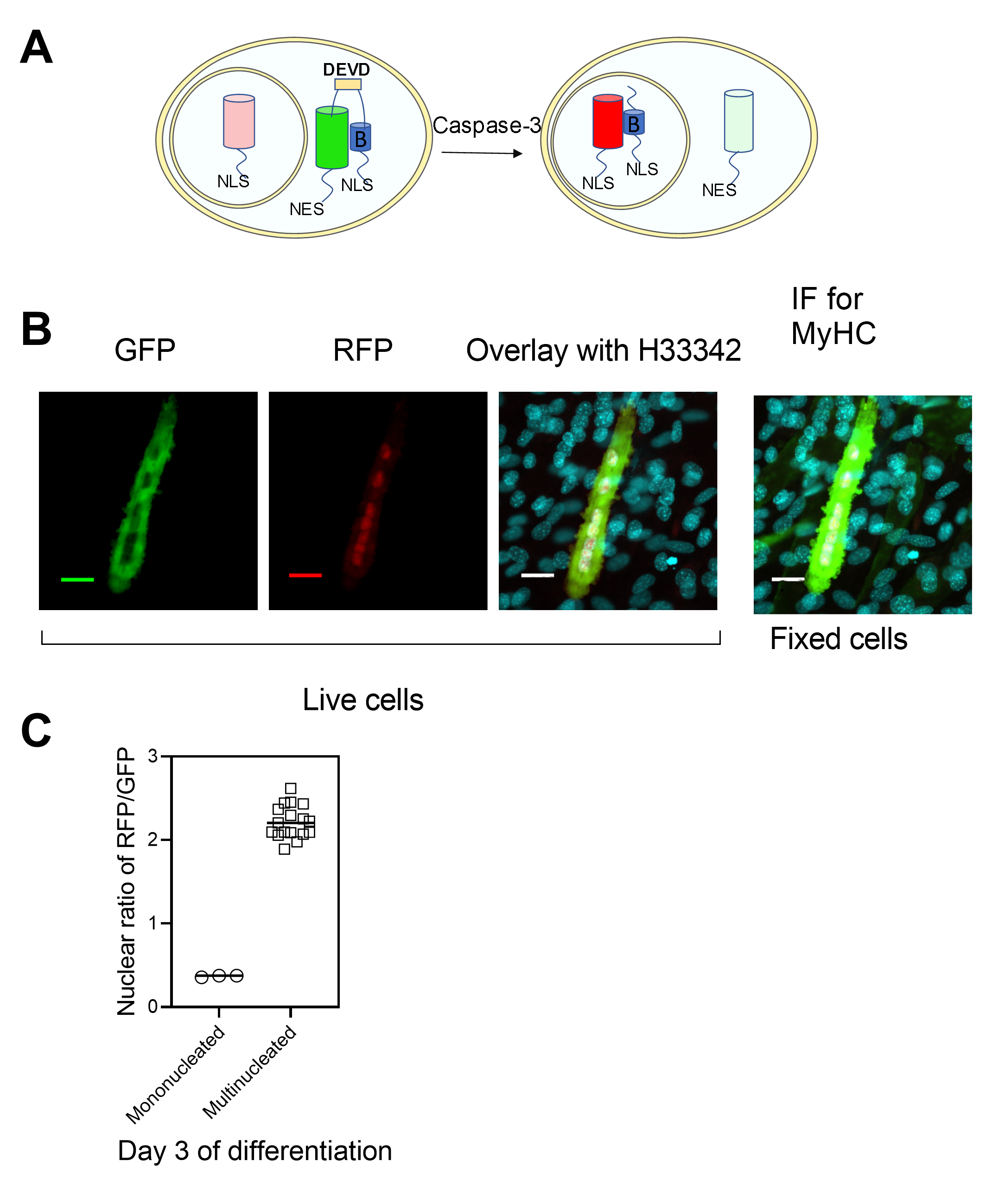

Supplement: Supplementary file 3 — Supplementary figure 2. [file 41419_2020_2502_MOESM3_ESM.tif]

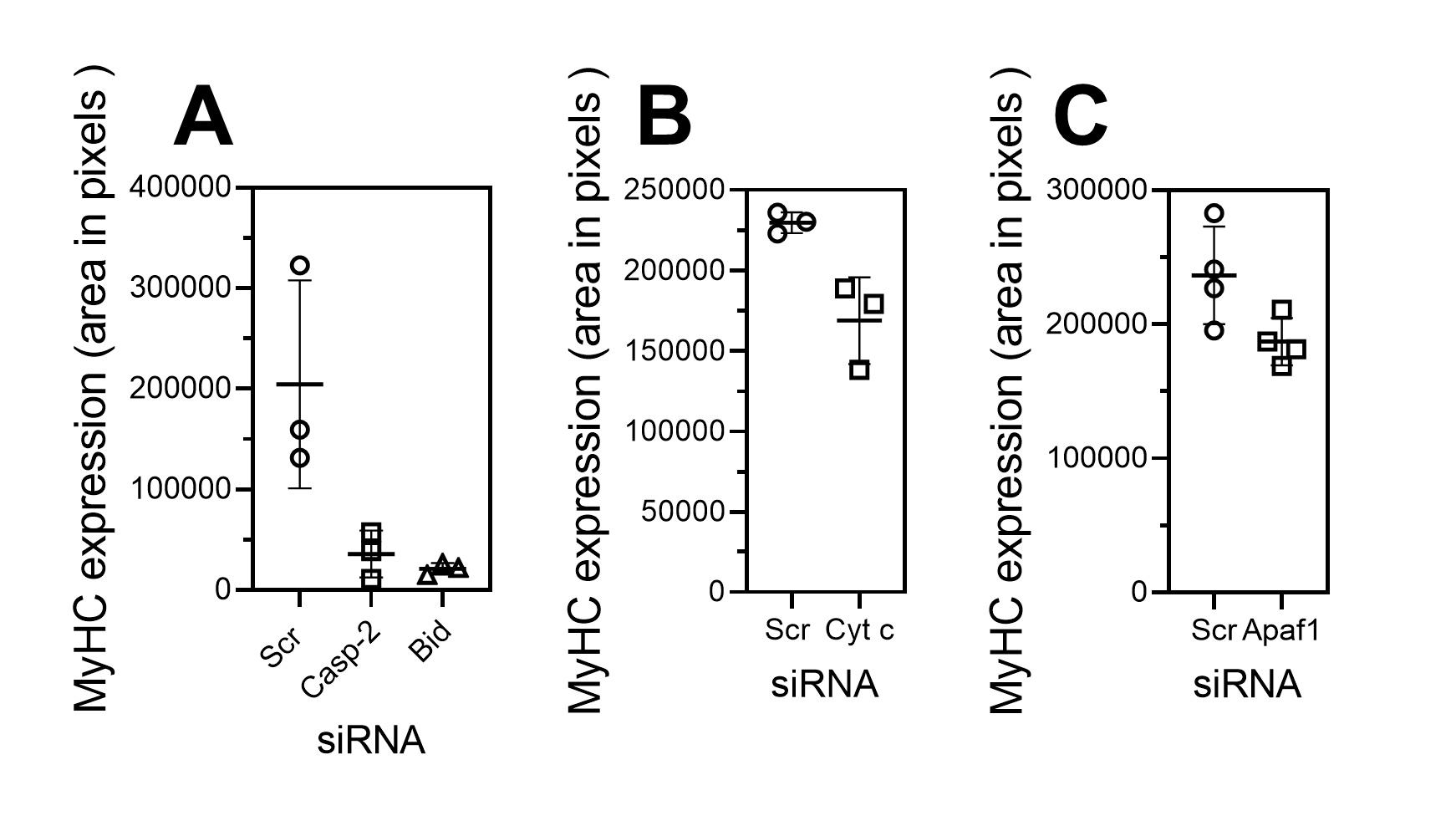

Supplement: Supplementary file 4 — Supplementary Figure 3 [file 41419_2020_2502_MOESM4_ESM.tif]

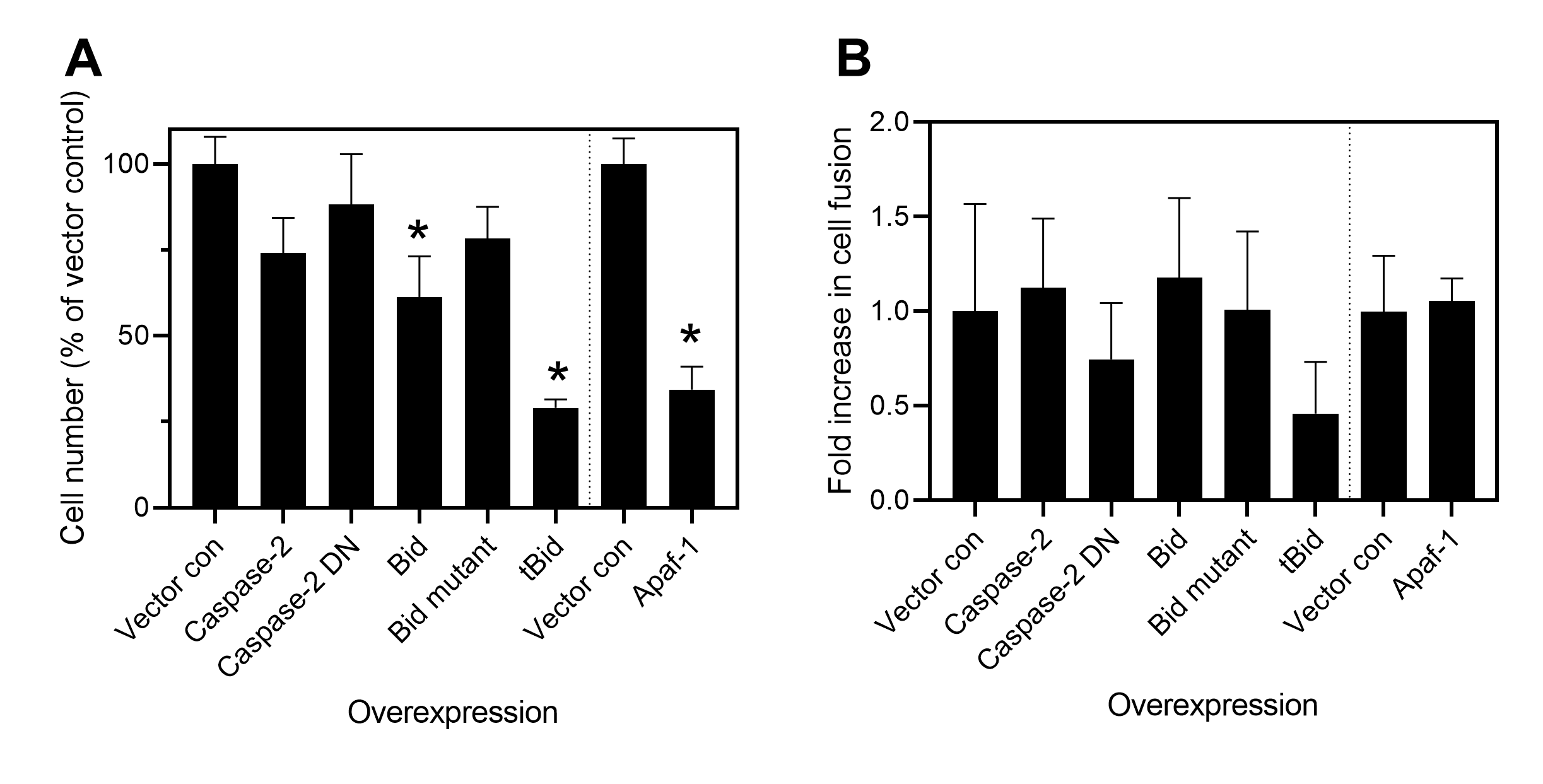

Supplement: Supplementary file 5 — Supplementary Figure 4 [file 41419_2020_2502_MOESM5_ESM.tif]
